# Burden of Stroke in China From 1990 to 2023: Analysis From the Global Burden of Disease Study 2023

**DOI:** 10.2196/86225

**Published:** 2026-06-22

**Authors:** Lijuan Fu, Wencai Jiang, Yanhua Peng, Xianjie Zhang, Rui Zhou

**Affiliations:** 1 Deyang People’s Hospital, Deyang, China; 2 Shanghai Fourth People's Hospital, No. 1279, Sanmen Road, Shanghai, China, 86 18108812438

**Keywords:** stroke, ischemic stroke, intracerebral hemorrhage, subarachnoid hemorrhage, burden of disease

## Abstract

**Background:**

China has initiated a national stroke program since 2011. Understanding the magnitude of stroke burden in China is crucial for modulating related policies.

**Objective:**

This study aimed to analyze stroke status in China from the 2023 Global Burden of Disease study.

**Methods:**

The 2023 Global Burden of Disease study was the source of the estimation of stroke burden. The data of deaths, incidence, prevalence, disability-adjusted life years (DALYs), years lived with disability (YLDs), and years of life lost attributable to stroke and its subtypes from 1990 to 2023 were analyzed. The mortality-to-incidence ratio was calculated to quantify the proportion of incident stroke cases that result in deaths. DALY decomposition analysis was applied to quantify how stroke burden has shifted between mortality and long-term disability over time. Sex-specific characteristics were also compared.

**Results:**

From 1990 to 2023, absolute stroke incidence (1.87-4.23 million) and prevalence (11.56-26.68 million) increased substantially, with ischemic stroke as the primary driver. Age-standardized incidence declined from 246.15 to 192.19 per 100,000 populations, while mortality significantly reduced from 302.72 to 92.83 per 100,000 populations. DALYs and years of life lost decreased 9.3% and 11%, respectively, while YLDs increased more than double. Mortality-to-incidence ratio fell for all subtypes except subarachnoid hemorrhage, indicating improved survival. Male individuals bore a heavier burden in total stroke and ischemic stroke, while subarachnoid hemorrhage showed female predominance in prevalence and YLDs. DALY decomposition revealed that YLDs contribution rose from 5.8% to 14.5%.

**Conclusions:**

From 1990 to 2023, despite declining age-standardized rates, stroke burden in China grew due to ischemic stroke and population changes. The shifting burden toward disability and persistent sex disparities highlights the need for tailored prevention, rehabilitation, and gender-specific interventions.

## Introduction

Stroke, encompassing ischemic stroke (IS), intracerebral hemorrhage, and subarachnoid hemorrhage (SAH), constitutes one of the leading causes of mortality, disability, and socioeconomic burden globally [1]. Characterized by acute onset, high morbidity, and long-term sequelae, stroke imposes profound challenges to health care systems and public health strategies worldwide [2]. In China, demographic transitions such as population aging; epidemiological shifts such as rising prevalence of hypertension, diabetes, and obesity; and changes in lifestyle factors have collectively shaped the landscape of stroke burden over the past three decades [3-5]. Understanding the temporal trends, subtype-specific patterns, and sex or age disparities in stroke incidence, mortality, prevalence, and disability-adjusted life years (DALYs) is critical for optimizing resource allocation, guiding preventive interventions, and evaluating the effectiveness of health care policies.

Over the past decades, China has experienced remarkable advancements in stroke prevention, acute care, and rehabilitation [4]. However, the absolute burden of stroke remains substantial due to population growth and aging [6]. Previous studies based on earlier iterations of the Global Burden of Disease (GBD) study have reported declining age-standardized mortality rates (ASMRs) for stroke in China, but the trends in incidence, prevalence, and DALYs—particularly across different stroke subtypes—remain incompletely characterized [7]. Moreover, the relative contributions of mortality-related burden (years of life lost [YLLs]) and disability-related burden (years lived with disability [YLDs]) to total DALYs have not been fully elucidated, which is essential for prioritizing interventions targeting either acute survival or long-term functional recovery.

The GBD Study 2021 provides the latest and most comprehensive estimates of global disease burden, incorporating updated data sources, improved statistical modeling, and expanded geographic coverage [8]. Leveraging this dataset, this study aims to (1) analyze the temporal trends in incidence, mortality, prevalence, DALYs, YLLs, and YLDs of total stroke and its major subtypes in China from 1990 to 2023; (2) explore sex-specific disparities in stroke burden; (3) evaluate changes in mortality-to-incidence ratio (MIR) to assess survival improvements; and (4) decompose DALYs to quantify the shifting contributions of YLLs and YLDs over time.

## Methods

### Data Source

This study was based on the GBD Study 2023, the latest version of GBD report [9]. Definitions of the diseases included by the GBD study are based on the International Classification of Diseases [10]. The details of data source and methodology of the GBD study can be accessible at the official website [11]. Data were extracted from the Global Health Data Exchange database established by the Institute for Health Metrics and Evaluation of America. Options in the search panel on this website were set up as follows: (1) GBD estimate: causes of death or injury; (2) measures: deaths, incidence, prevalence, DALYs, YLDs, and YLLs; (3) metric: number; (4) cause: stroke (IS, intracerebral hemorrhage, and SAH); (5) location: China; (6) age: all ages; (7) sex: both, male and female; and (8) year: years from 1990 to 2023. In addition, the age-standardized rate for incidence and death in 1990, 2021, and 2023 were also extracted.

### Data Processing

Data were presented as mean (95% uncertainty interval). The uncertainty levels were determined by the GBD modeling process as previously reported [12]. Values of lower and upper uncertainty can be extracted from the data source.

Burden of stroke, IS, intracerebral hemorrhage, and SAH were first analyzed. Sex-specific trends of the disease burden were consequently displayed respectively. The MIR was further calculated to quantify the proportion of incident stroke cases that result in death, avoiding complex population denominator dependencies. Lower MIR represents better survival (reflecting improved acute care, rehabilitation, or treatment efficacy).

DALY decomposition analysis was applied to decompose DALYs into its 2 core components—YLLs (mortality-related burden) and YLDs (disability-related burden)—to quantify how stroke burden has shifted between mortality and long-term disability over time.

### Ethical Considerations

This study did not include human or animal participants; therefore, ethics approval was not required for this study. For this type of study, formal consent was also not required.

## Results

### Disease Burden of Stroke in China From 1990 to 2023

**Figure 1.**
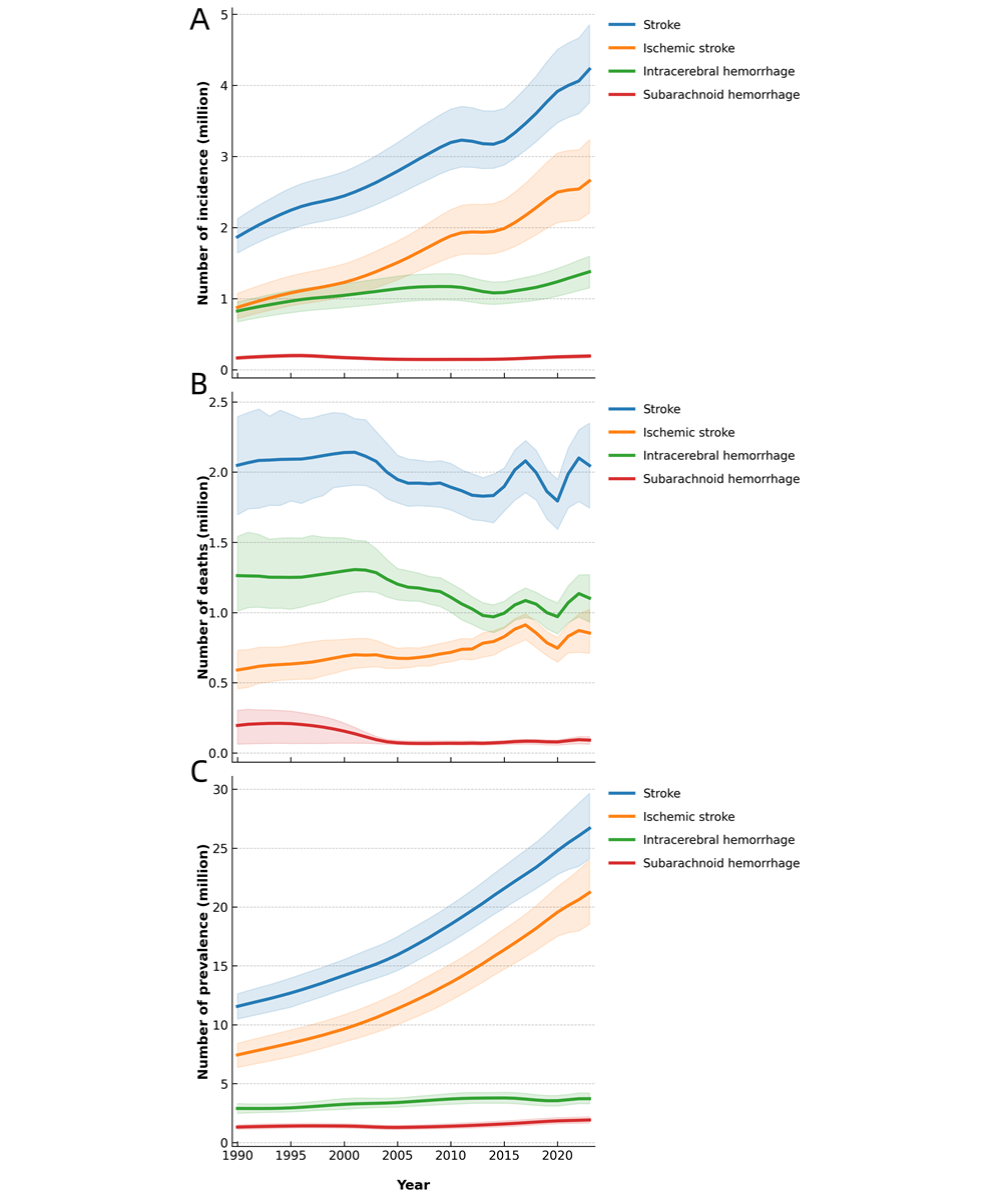
The trends of incidence (A), deaths (B), and prevalence (C) for stroke, ischemic stroke, intracerebral hemorrhage, and subarachnoid hemorrhage from 1990 to 2023.

**Figure 2.**
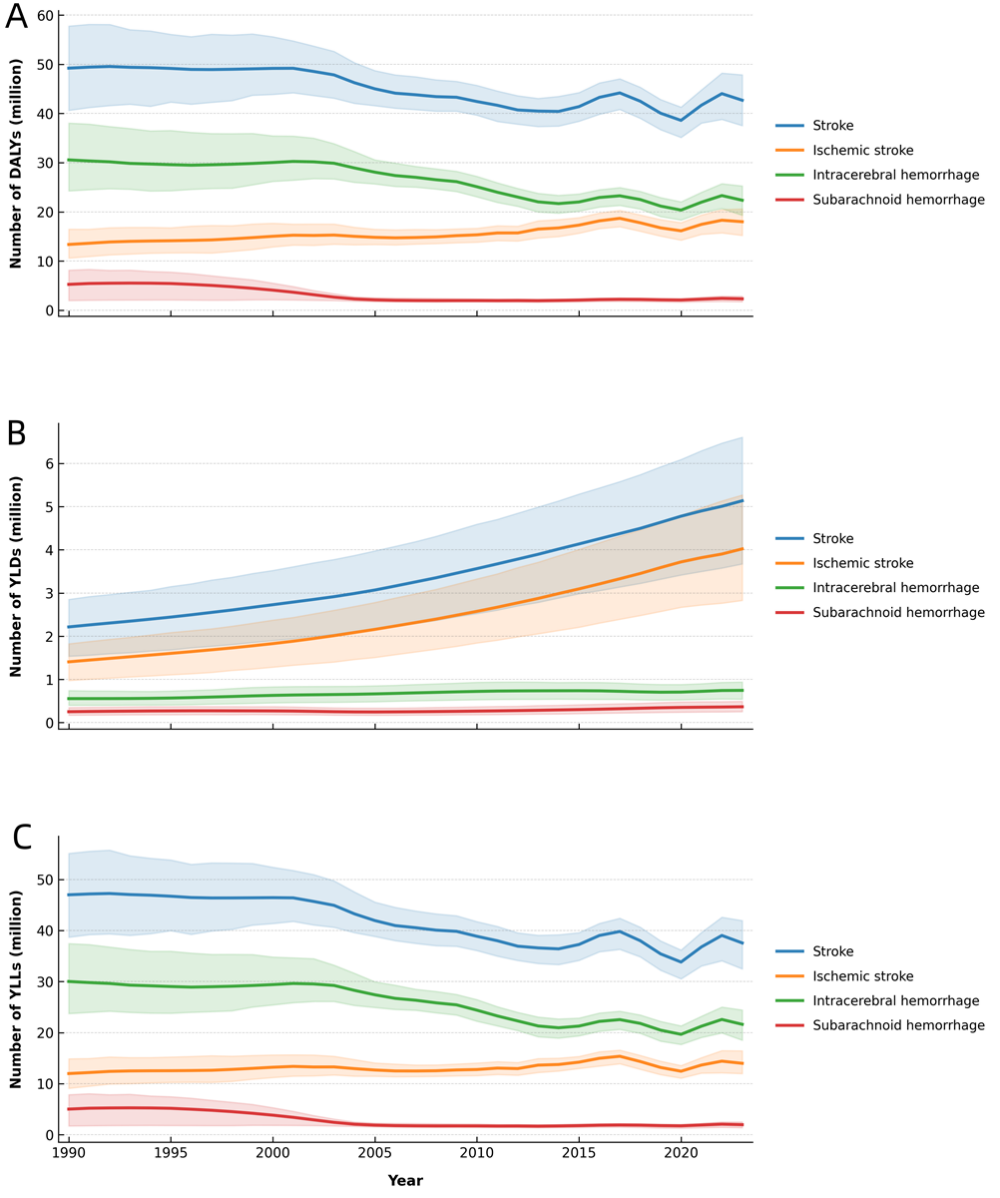
The trends of disability-adjusted life years (DALYs; **A**), years lived with disability (YLDs; **B**), and years of life lost (YLLs; **C**) for stroke, ischemic stroke, intracerebral hemorrhage, and subarachnoid hemorrhage from 1990 to 2023.

The absolute number of new stroke cases increased from 1.87 million in 1990 to 4.23 million in 2023 (+126%; Figure 1A). The age-standardized incidence rate (ASIR) declined from 246.15 per 100,000 population in 1990 to 192.19 per 100,000 in 2023 (–22%; Table 1). Total stroke deaths remained little changed from 1990 to in 2023 (Figure 1B), but the ASMR declined from 302.72 per 100,000 in 1990 to 92.83 per 100,000 in 2023 (–69%; Table 1). The total number of stroke patients dramatically increased from 11.56 million in 1990 to 26.68 million in 2023 (+131%; Figure 1C). From 1990 to 2023, the DALYs and YLLs decreased, but YLDs largely increased from 22.14 million to 51.33 million (Figure 2A-2C).

**Table 1.** The age-standardized rate of incidence and death for stroke, ischemic stroke, intracerebral hemorrhage, and subarachnoid in 1990, 2021, and 2023.

	Age-standardized incidence rate per 100,000 (95% UI)^a^			Age-standardized death rate per 100,000 (95% UI)		
	1990	2021	2023	1990	2021	2023
Stroke	246.15 (216.42-280.69)	193.54 (172.89-221.59)	192.19 (172.08-219.81)	302.72 (252.74-357.05)	97.48 (85.00-106.85)	92.83 (79.01-106.61)
Ischemic stroke	112.33 (93.74-135.06)	119.89 (100.86-143.77)	118.40 (100.04-141.31)	94.12 (74.19-116.98)	41.33 (35.21-45.99)	39.02 (32.35-46.75)
Intracerebral hemorrhage	114.44 (94.13-132.56)	64.01 (53.96-73.80)	64.16 (54.47-74.08)	181.68 (148.08-225.91)	51.96 (44.58-58.00)	49.72 (42.03-57.36)
Subarachnoid hemorrhage	19.39 (16.44-22.37)	9.63 (8.38-10.93)	9.63 (8.38-10.91)	5.69 (4.52-6.64)	4.19 (2.99-5.13)	4.10 (2.81-5.27)

a UI: uncertainty interval.

**Figure 3.**
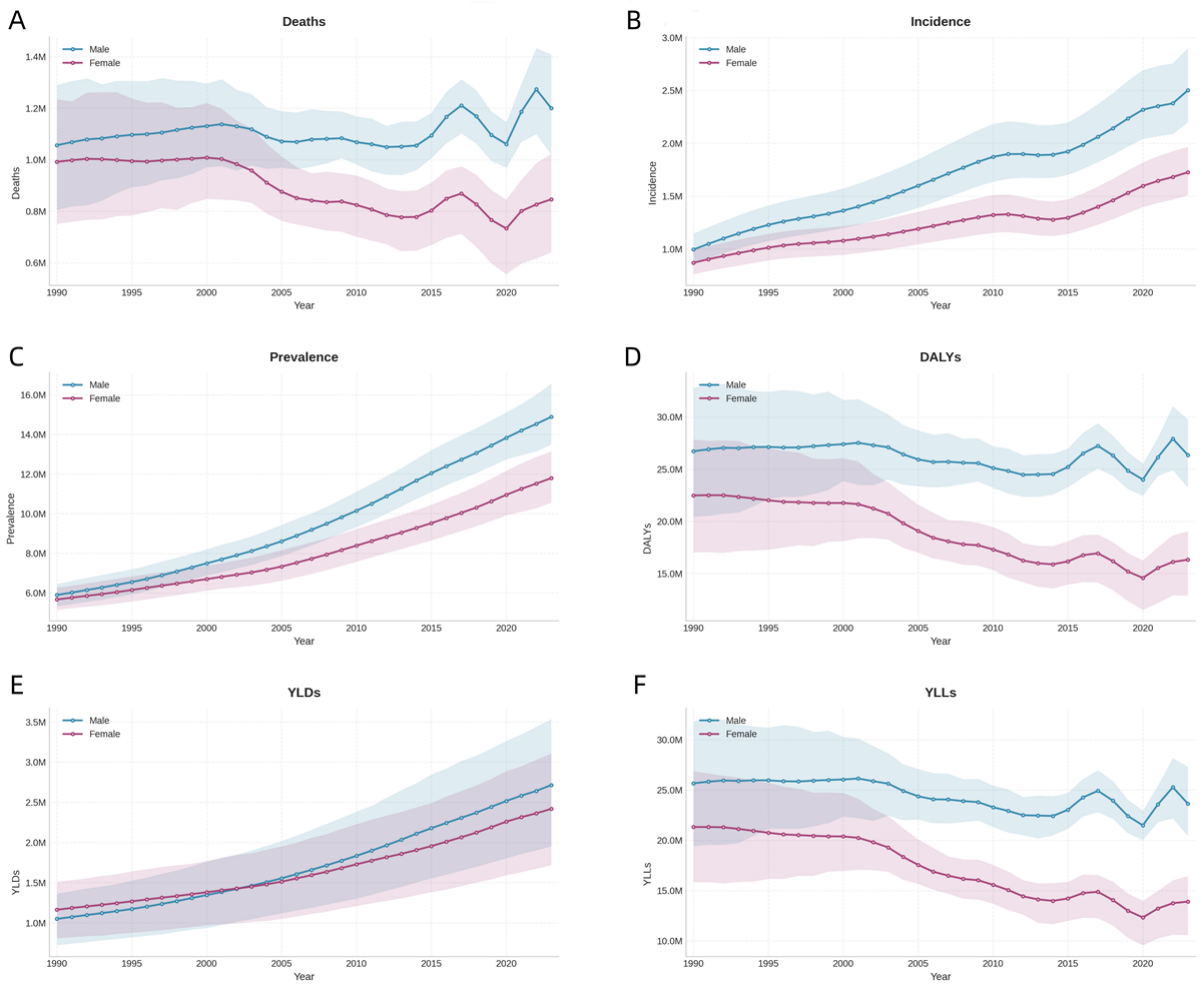
Gender-specific trends of stroke burden in China. DALY: disability-adjusted life year; YLD: years lived with disability; YLL: years of life lost.

A consistent male predominance in stroke burden was observed across all metrics throughout the study period, though the magnitude and trajectory of the gender gap varied by outcome (Figure 3A-3F). From 1990 to 2023, male stroke deaths slightly increased, while female deaths reduced (Figure 3A). Although the incidence, prevalence, and YLDs of both male and female increased, male individuals showed a larger increase in amplitude (Figure 3B, 3C, and 3E). DALYs and YLLs of male individuals slightly decreased, while female individuals had a significant reduction (Figure 3D and 3F).

### Disease Burden of IS in China From 1990 to 2023

The absolute number of new IS cases rose substantially from 0.88 million in 1990 to 2.66 million in 2023 (+202%; Figure 1A). Align with the growth in absolute incidence, the ASIR of IS also increased from 112.33 per 100,000 population in 1990 to 118.40 per 100,000 in 2023 (Table 1). Total IS deaths exhibited a rise over the study period (Figure 1B), but a significant ASMR plummeted from 94.12 per 100,000 in 1990 to 39.02 per 100,000 in 2023 (Table 1). The total number of people living with IS surged from 7.44 million in 1990 to 21.23 million in 2023 (Figure 1C). DALY-related metrics of IS all increased from 1990 to 2023, with YLDs showing a marked increase (Figure 2A-2C).

**Figure 4.**
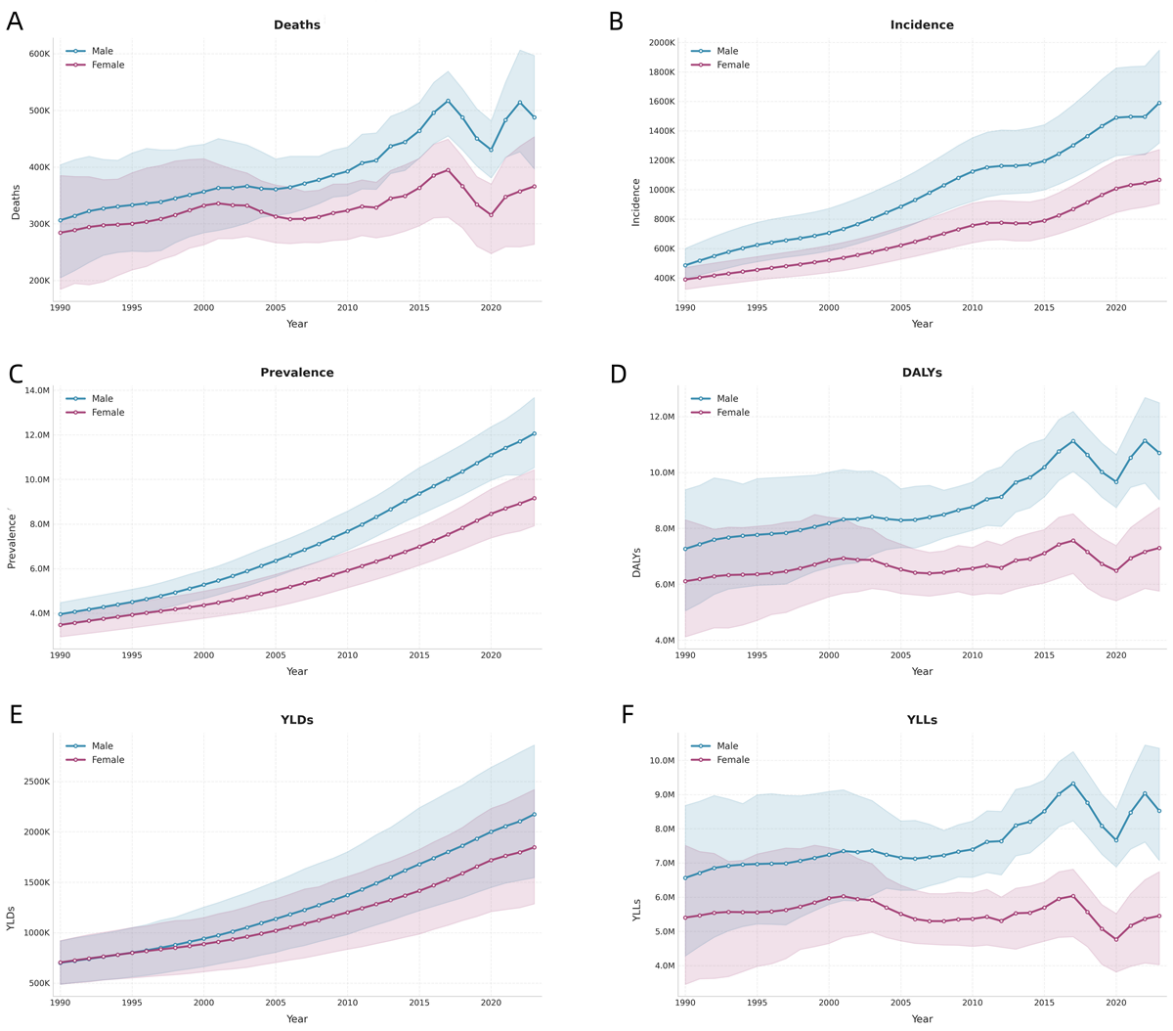
Gender-specific trends of ischemic stroke burden in China. DALY: disability-adjusted life year; YLD: years lived with disability; YLL: years of life lost.

Male predominance in IS burden was consistently increased across all metrics, though the size and trajectory of the gender gap differed by outcome (Figure 4A-4F).

### Disease Burden of Intracerebral Hemorrhage in China From 1990 to 2023

The incidence of intracerebral hemorrhage increased moderately from 0.83 million in 1990 to 1.38 million in 2023 (+66%; Figure 1A). In contrast to the rise in absolute incidence, the ASIR of intracerebral hemorrhage declined notably, falling from 114.44 per 100,000 population in 1990 to 64.16 per 100,000 in 2023 (−44%; Table 1). Total intracerebral hemorrhage deaths slightly decreased from 1990 to 2023 (Figure 1B), but ASMR showed a dramatic downward trend—dropping from 181.68 per 100,000 in 1990 to 49.72 per 100,000 in 2023 (–73%; Table 1). Benefiting from improved survival alongside mild incidence growth, the prevalence number rose from 2.89 million in 1990 to 3.72 million in 2023 (+29%; Figure 1C). Among DALY-related metrics, intracerebral hemorrhage DALYs and YLLs (mortality burden) both decreased, while YLDs (disability burden) increased moderately (Figure 2A-2C).

**Figure 5.**
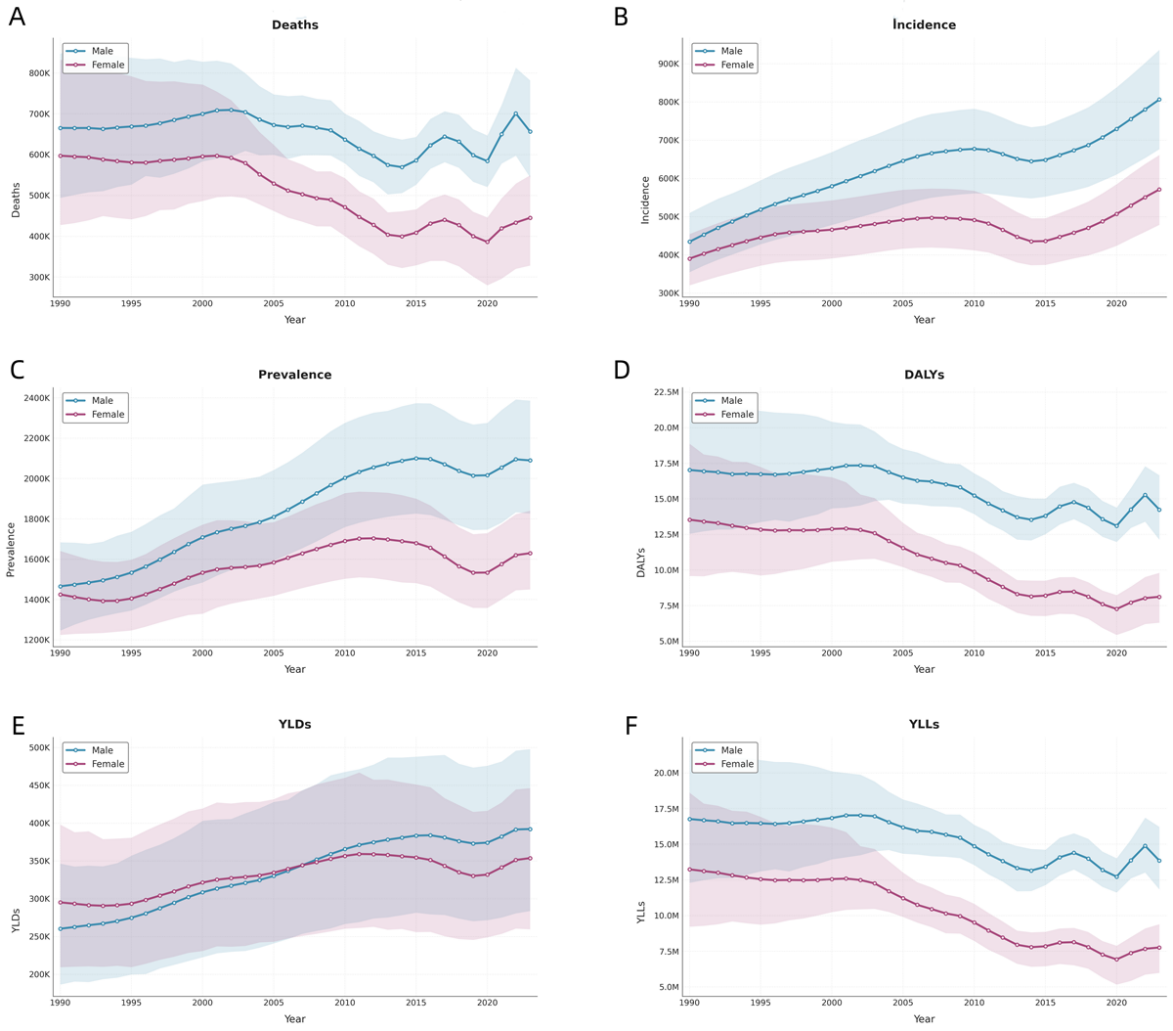
Gender-specific trends of intracerebral hemorrhage burden in China. DALY: disability-adjusted life year; YLD: years lived with disability; YLL: years of life lost.

Male predominance in intracerebral hemorrhage burden was consistent across all core metrics except YLDs (Figure 5A-5F). The YLDs of intracerebral hemorrhage in female individuals were higher than that in male individuals before 2007, while YLDs in male individuals have surpassed since 2008 (Figure 5E).

### Disease Burden of SAH in China From 1990 to 2023

The absolute number of new SAH cases increased slightly from 0.17 million in 1990 to 0.19 million in 2023 (Figure 1A). In contrast to the rise in absolute incidence, the ASIR of SAH showed a downward trend, falling from 19.39 per 100,000 population in 1990 to 9.63 per 100,000 in 2023 (−50%; Table 1). Total SAH deaths increased from 0.20 million in 1990 to 0.91 million in 2023 (+355%; Figure 1B), while the ASMR dropped slightly from 5.69 per 100,000 in 1990 to 4.10 per 100,000 in 2023 (−28%; Table 1). The total number of people living with SAH rose from 1.31 million in 1990 to 1.91 million in 2023 (Figure 1C). Among DALY-related metrics, SAH DALYs and YLLs both decreased notably, while YLDs increased moderately, expanding from 0.25 million in 1990 to 0.37 million in 2023 (Figure 2A-2C).

**Figure 6.**
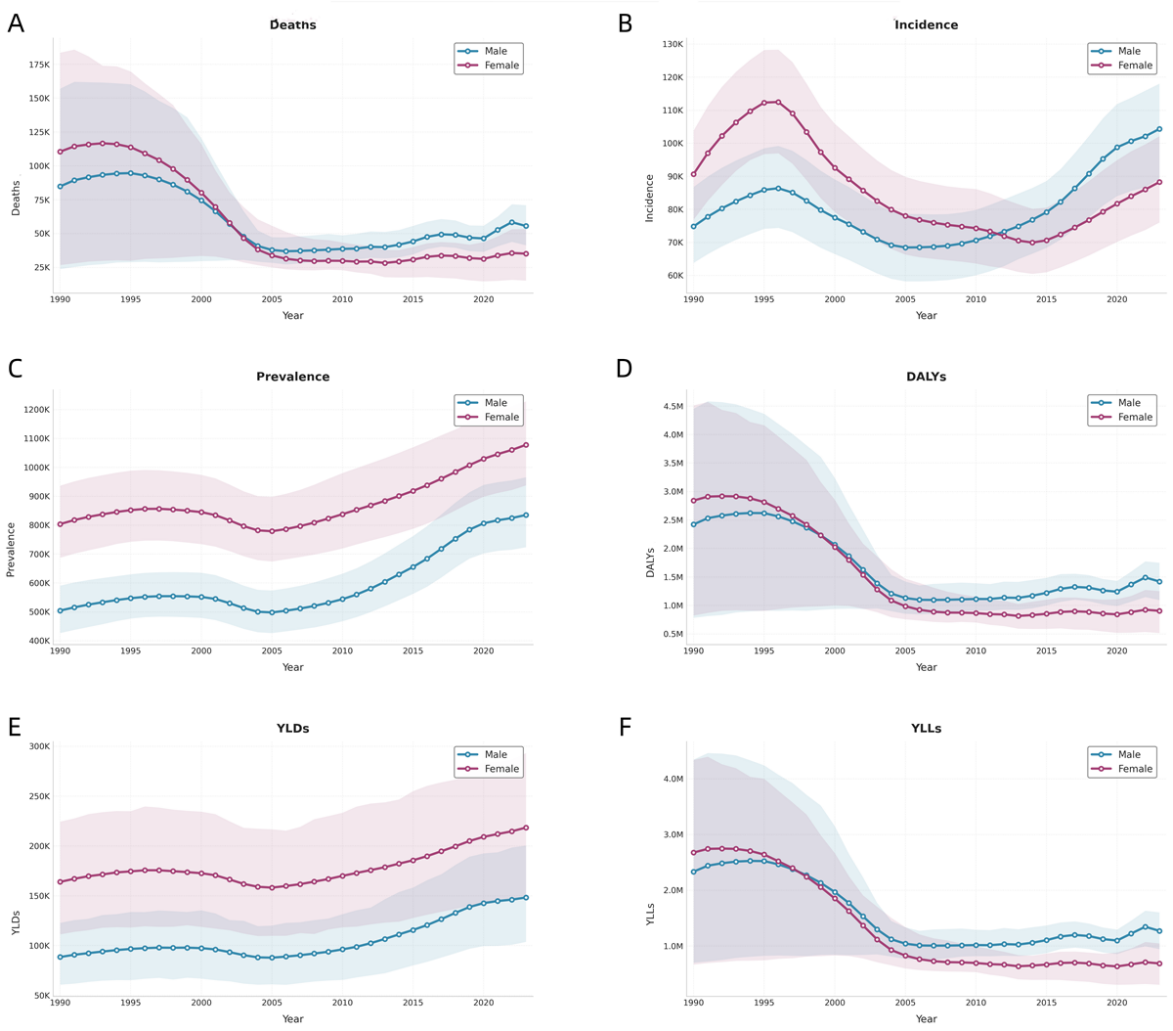
Gender-specific trends of subarachnoid hemorrhage burden in China. DALY: disability-adjusted life year; YLD: years lived with disability; YLL: years of life lost.

Unlike other subtypes of stroke, the prevalence and YLDs of female were larger than that of male individuals during the study period (Figure 6C and 6E). The incidence, deaths, DALYs, and YLLs of female individuals were once higher than that of male individuals but have been surpassed (Figure 6A, 6B, 6D, and 6F).

### Analysis of MIR

All stroke types except SAH showed reduced MIR from 1990 to 2023 (Table 2). Intracerebral hemorrhage had the highest MIR in both years, while IS showed a largest reduction rate (Table 2). In 1990, male individuals had higher MIR than female individuals for all subtypes, while the MIR in 2023 was opposite except SAH (Table 2).

**Table 2.** Mortality-to-incidence ratio of stroke and its subtypes in 1990 and 2023.

	Total (95% UI)^a^	Male (95% UI)	Female (95% UI)
Stroke			
1990	1.23 (1.07‐1.42)	1.27 (1.09‐1.48)	1.19 (1.02‐1.39)
2023	0.48 (0.42‐0.56)	0.46 (0.40‐0.53)	0.51 (0.44‐0.59)
Ischemic stroke			
1990	0.84 (0.69‐1.03)	0.87 (0.71‐1.07)	0.81 (0.65‐1.01)
2023	0.33 (0.28‐0.40)	0.31 (0.26‐0.38)	0.35 (0.29‐0.43)
Intracerebral hemorrhage			
1990	1.59 (1.34‐1.89)	1.64 (1.37‐1.96)	1.54 (1.28‐1.85)
2023	0.77 (0.66‐0.91)	0.74 (0.63‐0.88)	0.80 (0.68‐0.95)
Subarachnoid hemorrhage			
1990	0.29 (0.23‐0.37)	0.31 (0.24‐0.40)	0.28 (0.21‐0.36)
2023	0.43 (0.33‐0.57)	0.45 (0.34‐0.60)	0.41 (0.31‐0.55)

a UI: uncertainty interval.

### Decomposition Analysis of Stroke DALYs

Decomposition of stroke DALYs is shown in Table 3. For the total population, DALY composition shifted uniformly from mortality to disability between 1990 and 2023: YLLs contribution declined across all subtypes while YLDs contribution nearly tripled for total stroke (5.8%-14.5%) and doubled for SAH (8.3%-17.1%), with IS becoming the most disability-heavy subtype. Male individuals consistently had higher YLLs contribution and lower YLDs contribution than female individuals. Female individuals, by contrast, had the steepest YLLs decline and fastest YLDs growth, driving the total population’s disability shift and widening the sex gap in YLDs contribution from 2% to 6.7% for total stroke. Intracerebral hemorrhage remained mortality-dominant (YLLs ≥95.5% in 2023) across all groups, though female individuals still had slightly higher YLDs contribution than male individuals for this subtype.

**Table 3.** Decomposition of stroke disability-adjusted life years into years of life lost (YLLs) and years lived with disability (YLDs) by sex in 1990 and 2023.

	Total population		Male		Female	
	YLLs contribution, % (95% UI)^a^	YLDs contribution, % (95% UI)	YLLs contribution, % (95% UI)	YLDs contribution, % (95% UI)	YLLs contribution, % (95% UI)	YLDs contribution, % (95% UI)
Stroke						
1990	94.2 (93.5‐95.0)	5.8 (5.0‐6.5)	95.1 (94.3‐95.8)	4.9 (4.2‐5.7)	93.1 (92.2‐94.0)	6.9 (6.0‐7.8)
2023	85.5 (84.3‐86.7)	14.5 (13.3‐15.7)	88.8 (87.5‐90.0)	11.2 (10.0‐12.5)	82.1 (80.6‐83.6)	17.9 (16.4‐19.4)
Ischemic stroke						
1990	92.6 (91.7‐93.5)	7.4 (6.5‐8.3)	93.5 (92.5‐94.4)	6.5 (5.6‐7.5)	91.5 (90.3‐92.7)	8.5 (7.3‐9.7)
2023	77 (75.6‐78.4)	23(21.6‐24.4)	79.7 (78.0‐81.4)	20.3 (18.6‐22.0)	74.1 (72.2‐76.0)	25.9 (24.0‐27.8)
Intracerebral hemorrhage						
1990	97.1 (96.4‐97.8)	2.9 (2.2‐3.6)	97.5 (96.8‐98.1)	2.5 (1.9‐3.2)	96.7 (95.9‐97.5)	3.3 (2.5‐4.1)
2023	96.2 (95.4‐97.0)	3.8 (3.0‐4.6)	96.8 (96.0‐97.5)	3.2 (2.5‐4.0)	95.5 (94.5‐96.5)	4.5 (3.5‐5.5)
Subarachnoid hemorrhage						
1990	91.7 (90.2‐93.1)	8.3 (6.9‐9.8)	92.4 (90.7‐94.0)	7.6 (6.0‐9.3)	90.9 (89.0‐92.7)	9.1 (7.3‐11.0)
2023	82.9 (80.8‐85.0)	17.1 (15.0‐19.2)	`85.3 (82.8‐87.8)	14.7 (12.2‐17.2)	80.4 (77.8‐83.0)	19.6 (17.0‐22.2)

a UI: uncertainty interval.

## Discussion

In general, the burden of stroke increased from 1990 to 2023 in China, despite the decline of ASIR and mortality rate. Our study showed that the overall deaths, incidence, and prevalence increased by 11.8%, 38.2%, and 130.8%, respectively, while DALYs and YLLs decreased by 9.3% and 11%, respectively. Our results were based on the estimated number, which is different from the previous study [13]. The reason why we chose the estimated number as the metric is that the increasing number of stroke patients is generally associated with increasing financial burden and consumption of medical resources. Thus, the increase of stroke-related burden should be attached much more attention.

Specifically, the increase mainly came from IS, though the burden of intracerebral hemorrhage and subarachnoid also slightly increased. IS, primarily caused by embolism and arterial atherosclerotic disease, often presents as sudden-onset unilateral limb paralysis, facial asymmetry, abnormal speaking, confusion, changes of consciousness, unbalance or incoordination, and visual impairment [14,15]. As well recognized, hypertension, smoking, hyperlipemia, diabetes, transient ischemic attacks, and atrial fibrillation are the common risk factors for IS. A previous study based on 15 nationwide surveys pointed out that the prevalence of hypertension in China was 18% to 44.7% [16]. A secondary study from a national cross-sectional study showed that 2.31% of the enrolled 0.7 million participants had atrial fibrillation [17]. Moreover, metabolic factors were more severe, with an estimated morbidity of 31% and 38% for hyperlipemia and diabetes, respectively [18,19]. In addition, as the largest producer and consumer of cigarettes in the world, the smoking rate was estimated to be over 20% in China [20]. All these risks contributed to the increased incidence of IS. However, the mortality of IS has declined significantly over the past 30 years.

Progresses have been made to improve the survival of IS, including the treatment and prevention strategies. Of most importance, initiation of thrombolysis within 4.5 hours and endovascular thrombectomy within 24 hours after symptom onset are considered to improve neural outcomes and are recommended by the guidelines [21-23]. The composition of thrombolytic drugs (Tenecteplase, etc) and anesthesia methods (procedural sedation and general anesthesia) have also been investigated and applied to acute IS [24,25]. The whole cycle management of IS advocates early detection and reperfusion, and a multidisciplinary care in hospital and out of hospital. In this study, the MIR significantly decreased from 1990 to 2023, indicating great improvement in stroke care. All these advances, consequently, augment the burden of DALYs and YLDs. Our results of DALYs and YLDs follow this rule. Another vital policy of China is to establish stroke centers, where the stroke patients can be treated as soon as possible in a standardized procedure [26]. Stroke centers are correlated to better outcomes of patients with acute IS [27,28].

These 34-year data on stroke burden in China reveal notable sex-specific patterns across total stroke and its subtypes, driven by biological differences, gendered risk factor exposure, and health care access, which warrant targeted public health attention. A notable point is the widening gaps between male and female individuals in the burden of stroke—a disease highly related to lifestyle [29,30]. In China, male individuals had higher rates of smoking and heavy alcohol use [31,32].

When interpreting the results, we should first understand the intrinsic limitations of the GBD study. As described, the estimated disease burden is based on the availability of original data and calculation models, which makes it difficult to fully represent the actual situation [12]. The inherent uncertainties in GBD estimations and potential reliance on limited or low-quality data for certain inputs should also be noticed. Nevertheless, the GBD study is the most reliable tool to evaluate global health issues. Secondly, the GBD study does not provide province-level data, which is important to understand the regional characteristics. Finally, the study does not account for the impact of potential confounding factors on stroke burden trends, such as the contributions of specific modifiable risk factors (eg, regional variations in hypertension control rates, smoking prevalence, or dietary patterns) or nonmodifiable factors (eg, genetic susceptibility) to the observed trends.

In conclusion, from 1990 to 2023, stroke burden in China saw growing absolute numbers despite declining ASIRs and mortality rates, driven primarily by IS. The burden shifted toward disability as evidenced by the decreased MIR and increased YLDs’ contribution to DALYs. Male individuals faced heavier stroke burden. These trends call for tailored strategies: IS prevention, enhanced rehabilitation for disability, and gender-specific interventions alongside sustained investment in stroke care systems.

## Abbreviations

ASIR: age-standardized incidence rate
ASMR: age-standardized mortality rate
DALY: disability-adjusted life year
GBD: Global Burden of Disease
IS: ischemic stroke
MIR: mortality-to-incidence ratio
SAH: subarachnoid hemorrhage
YLDs: years lived with disability
YLLs: years of life lost
